# Association between elastic and muscular artery stiffness and organ dysfunction in patients with early severe sepsis

**DOI:** 10.1186/2197-425X-3-S1-A643

**Published:** 2015-10-01

**Authors:** S Kazūne, A Grabovskis, E Strīķe, I Vanags

**Affiliations:** Department of Anaesthesiology, Hospital of Traumatology and Orthopaedics, Riga, Latvia; Department of Anaesthesiology and Reanimatology, Riga Stradins University, Riga, Latvia; Institute of Atomic Physics and Spectroscopy, University of Latvia, Riga, Latvia; Department of Anaesthesiology and Cardiac Surgery, Pauls Stradins Clinical University Hospital, Riga, Latvia

## Introduction

Sepsis is characterised by massive inflammatory response, which can affect vascular function. As part of vascular dysfunction large arteries can be affected. To what extent changes in large artery function contribute to organ dysfunction in sepsis is not well studied.

## Objectives

We investigated carotid to femoral and carotid to radial pulse wave velocity (PWV) as an index of sepsis-induced changes in elastic and muscular arteries, and the association of changes in PWV with subsequent change in the SOFA score.

## Methods

Consecutive patients hospitalised to the 12-bed Toxicology and Sepsis Clinic of Riga East Clinical University Hospital with a diagnosis of severe sepsis or septic shock were enrolled within 24 hours of admission. Baseline carotid to femoral and carotid to radial PWV were determined at enrollment and after 48 hours of initial measurement. Patients showing an improved SOFA score at 48 hours (SOFA improvers) were compared to those for whom it was unchanged or worse (SOFA non-improvers).

## Results

Of the 37 patients, 27 patients were SOFA improvers and 10 SOFA non-improvers. They had comparable APACHE II scores (18 (15-23.5) vs 19 (12.5-27)) on admission. SOFA improvers had lower baseline carotid to radial PWV values (10.4 (8.4-16.2) vs. 13.7 (6.9-14.9) m/s, p = 0.02) and less increase of carotid to radial PWV after 48 hours (49.9 % (-29% to 61.2%) vs -16.5%(-37.4% to 18.7%), p = 0.008) compared to the SOFA non-improvers. Carotid to radial PWV values similarly correlated with the intra-hospital mortality. Carotid to femoral PWV was similar between the two groups of patients (11.9 (9-16.5) vs 10.1 (8.2-14.4) m/s).

## Conclusions

PWV in elastic arteries is similar in patients who had an improvement in organ failure in the first 48 hours and those who had unchanged or worse condition. In contrast, PWV in muscular arteries is lower and tends to increase over the first 48 hours in SOFA improvers and in patients with favourable outcome.

